# Comprehensive Analysis of the Copper Exchange Implemented in Ammonia and Protonated Forms of Mordenite Using Microwave and Conventional Methods

**DOI:** 10.3390/molecules24234216

**Published:** 2019-11-20

**Authors:** Marina G. Shelyapina, Ekaterina A. Krylova, Yurii M. Zhukov, Irina A. Zvereva, Inocente Rodriguez-Iznaga, Vitalii Petranovskii, Sergio Fuentes-Moyado

**Affiliations:** 1 Saint-Petersburg State University, 7/9 Universitetskaya nab., St. Petersburg 199034, Russia; krylovaea2803@mail.ru (E.A.K.); yuri.m.zhukov@gmail.com (Y.M.Z.); irinazvereva@yandex.ru (I.A.Z.); 2 Instituto de Ciencias y Tecnología de Materiales (IMRE)–Universidad de La Habana, Zapata y G, s/n La Habana 10400, Cuba; inocente@imre.uh.cu; 3 Centro de Nanociencias y Nanotecnología, Universidad Nacional Autonoma de Mexico, Ensenada 22860, Baja California, Mexico; vitalii@cnyn.unam.mx (V.P.); fuentes@cnyn.unam.mx (S.F.-M.)

**Keywords:** mordenite, microwave-assisted ion exchange, XRD, ICP-OES, NMR, TGA, XPS

## Abstract

This article presents the results of a comprehensive study of copper-exchanged mordenite samples prepared from its ammonia and protonated forms (Si/Al = 10) using two different ion exchange methods: conventional and microwave (MW)-assisted. The protonated H-MOR-10 sample was obtained by calcination of commercial NH_4_-MOR-10; in this case, a slight degradation of the mordenite framework was observed, but the resulting defects were partially restored after the first ion-exchange procedure of protons for copper ions. The level of copper exchange in the studied materials was found to be limited to 70%. Regardless of the exchange procedure, the replacement of ammonium or proton ions with copper led to a linear increase in the *a*/*b* ratio of cell parameters in accordance with an increase in the level of copper exchange, which means that all Cu^2+^ cations are ion-exchangeable and enter the main mordenite channel. Thermal analysis indicated a correlation between the replacement of various ammonium and hydroxyl groups by copper ions during the exchange treatment and their dehydroxylation energy during thermal decomposition. As a conclusion: MW-assisted treatment proved itself as an efficacious method for the synthesis of copper-exchanged mordenites, which not only significantly reduces preparation time but leads to a systematically higher copper exchange level.

## 1. Introduction

Zeolites have unique structural and chemical properties that are vital for a wide range of industries. Given their economic effect, there is a powerful incentive for the intelligent design of new materials with enhanced functionality in ion exchange and catalysis [1]. Cation-exchanged zeolites are a prospective class of catalysts and their popularity has steadily increased over the last years. Following the study of copper-containing zeolites as promising materials for the removal of nitrogen oxides from exhaust gases (de-No*x* catalysts) [2,3,4,5], new processes based on ion-exchanged zeolites have recently been investigated. Zeolites, as the most important solid catalysts used in traditional petrochemical industries, also find prospective applications in many sustainable processes. In addition, they can be used to capture and convert CO_2_ in fuel cells, for biomass conversion, air, and water purification, etc. [6,7,8].

Among the many known zeolites, mordenite is one of the most interesting, from our point of view. It is one of the most used zeolites in the chemical industry and plays a key role in novel applications. The reason for this is that this zeolite belongs to the so-called “big five” zeolites, which are extensively applied in the industry as catalysts [9]. Based on this, the constant interest in a detailed study of this material is quite understandable.

To focus on one of the possible systems for research, choosing the metal to modify mordenite was necessary. Among the proposed metals, for several reasons, we considered copper to be one of the most interesting. First, from an economic point of view, it has an acceptable price. Secondly, copper, an element with a variable valence and with a very interesting, although not fully investigated, chemistry on the surface of zeolitic pores [10], has repeatedly shown very fascinating results in various processes. Numerous studies have shown that the catalytic properties of copper-exchanged zeolites are determined by the valence state of copper ions and their location and coordination in the zeolite lattice, as well as by the copper content [10,11]. According to Iwamoto [12], the so-called over-exchanged zeolites (i.e., zeolites containing more polyvalent cations than is necessary for formal charge equilibrium) are more efficient in catalytic reactions. From this point of view, it is very important to find an effective method for controlling the copper content in the zeolite matrix.

It has been recently discovered [13] that extra-framework aluminum species can also affect the catalytic activity of Cu-mordenite in the conversion of methane to methanol. Moreover, the materials obtained from a protonated form have been found to be more active. As it has been shown, there are two key properties that determine the activity of prepared materials in methane conversion: the parent counterion and the copper ion-exchange procedure [13].

It is well known that the performance of a Cu-zeolite catalyst strongly depends on the chemical state of copper. In turn, it is accepted that the ion exchange method affects the appearance of Cu species; the matrix topology and counterions presented in the starting zeolite also influence this appearance [14]. The recent development of microwave-assisted treatment methods has introduced a new direction in the development of chemistry of materials [15,16,17,18,19,20]. Being a simple, inexpensive, and efficient nonconventional heating method, the microwave (MW) technique is well established in the field of alternative methods for the synthesis of emerging materials, including zeolites [17,19,20,21,22]. A comparison of several Ni-mordenites obtained by ion exchange of Na-mordenite in the liquid phase under irradiation by microwaves and by the conventional method showed that after the first exchange an analogous amount of exchangeable Ni^2+^ ions, with and without MW assistance, was achieved [21], but was faster with MW irradiation. Interestingly, microwaves strongly affected the acidity of the samples obtained, the acidity of which increased with longer treatment with microwaves due to the appearance of new weak acid sites. These new centers were not observed for the sample exchanged in the conventional way [21]. Differences in the acidity of samples prepared by conventional and microwave-assisted treatments were also observed in [22].

Finally, the effect of counter-cations in the initial zeolites can change the final properties of the obtained samples. In [23], copper-ammonium exchange was studied for the ammonium form of SAPO zeolites obtained by various methods. A number of NH_4_-SAPO-34 samples prepared by NH_3_ adsorption were prepared and used for comparison with NH_4_-SAPO-34 obtained by conventional exchange from a liquid. Even if an analogous amount of the NH_4_^+^ ion was contained in the sample obtained by gas adsorption and in the sample obtained by the liquid ion exchange method, there was still a difference between the coordination of NH_4_^+^ species with the bridging hydroxyl groups of Si(OH)Al. This difference resulted in a subsequent exchange of Cu, leading to various forms of cationic copper complexes [23].

Earlier, we analyzed the influence of various factors in the preparation of copper exchange mordenites on the properties of the materials obtained [24,25,26,27,28]. In [27], we reported the results of our comprehensive study of two sets of copper-exchanged samples of mordenites prepared from the sodium form of this zeolite and obtained by two different methods: conventional and MW assisted. It was found that the MW-assisted procedure resulted in a deeper and even over-exchange. The main goal of present study was to prove the ability and efficacy of MW-assisted treatment, as compared with the conventional procedure, in synthesis of copper-exchanged mordenite from ammonia and protonated forms.

## 2. Results

Throughout the text, tables, and figures, the samples obtained from the ammonia and protonated forms are labeled as CuNH_4_-MOR-10-*XY* and CuH-MOR-10-*XY*, respectively, where *X* = C or M to represent the conventional (C) or MW (M) ion exchange preparation method; *Y* = number of copper-exchange procedures; digit 10 corresponds to the Si/Al ratio. For more details, see Section 4. Materials and Methods.

### 2.1. Elemental Analysis

Table 1 shows the Si/Al and Cu/Al ratios for all the studied samples, determined using inductively coupled plasma-optical emission spectroscopy (ICP-OES). These numbers were calculated on the basis of real AR values and assumed that all copper ions were in Cu^2+^ state (the copper state was confirmed by the XPS results, see further). In the last column of Table 1 the copper exchange level is given, which is the percentage of copper in the sample relative to the maximum possible amount of Cu^2+^ cations if all NH_4_^+^ or H^+^ cations that compensate for the charge in the mordenite structure are fully exchanged.

It should be noted that the bulk ICP-OES analysis of the starting NH_4_-MOR-10 sample gave an Si/Al atomic ratio (AR) equal to 8.3 instead of that stated by Zeolist Int. (AR equal to 10.0). A similar effect of the lower real AR (5.9 instead of 6.5) was observed for the earlier studied sample Na-MOR-6.5 [27], supplied by the same fabricator. As this material was ordered from an industrial manufacturer, their method of composition analysis, permissible deviations from the nominal values, and reproducibility of sequential routine syntheses are unknown. Since the protonated form (H-MOR-10) was obtained by calcination of the NH_4_-MOR-10 mordenite, its AR was the same, as expected.

### 2.2. Structural Analysis

**Figure 1:**
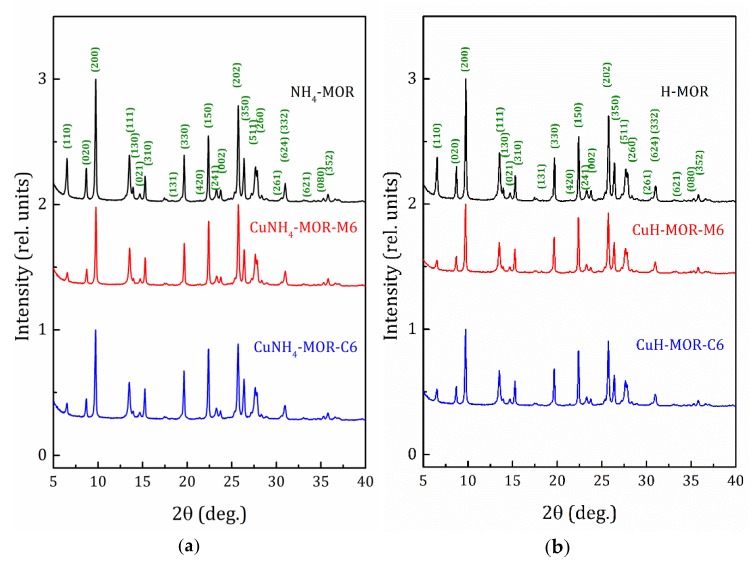
X-ray diffraction (XRD) patterns for the starting and copper-exchanged samples obtained after six MW-assisted and conventional procedures; (**a**) -ammonia form and (**b**) -proton form.

X-ray diffraction (XRD) patterns of the NH_4_-MOR-10 and H-MOR-10 samples, as well as the patterns for the copper-exchanged samples, obtained after six conventional and MW-assisted ion-exchange procedures, are shown in Figure 1. The XRD patterns for all the studied samples can be found in Supplementary Materials (Figures S1 and S2). The XRD analysis confirmed that all the samples kept the mordenite crystalline structure and no peaks corresponding to other phases were observed. The line width analysis, done by applying the well-known Scherrer formula, revealed that the average crystallite size for all the studied samples was about 50–60 nm.

The lattice parameters for the studied samples before and after ion-exchange procedures are listed in Table 2. Ion-exchange treatment led to slight changes in unit cell parameters, keeping the unit cell volume almost unchanged. In general, the main tendency in the lattice parameters changed was the following: the exchange of ammonia (or proton) cation for copper resulted in expansion along the *a*-axis and was accompanied by a simultaneous compression along the *b*-axis. The lattice parameter along the *c* axis remained almost unchanged. This means that the deformation of the mordenite crystal lattice occurred in a plane perpendicular to the *c*-axis. A similar, but more pronounced, effect was observed for the Na-series of Cu-exchanged mordenites [27]. For a better description of this lattice deformation upon copper loading we introduced the contraction parameter *K* = *a*/*b*, which is also listed in Table 2 and will be discussed further.

**Figure 2:**
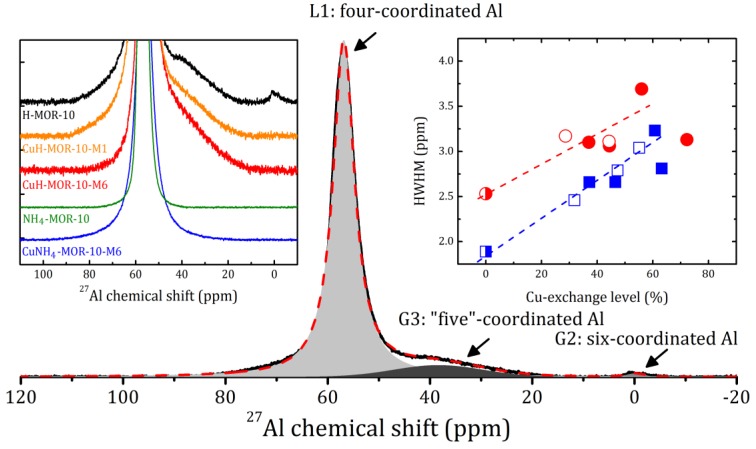
^27^Al MAS NMR spectrum and its decomposition on one Lorentzian (L1) and two Gaussian (G2 and G3) lines for H-MOR-10 at rotation frequency 8 kHz. Left insert: ^27^Al MAS NMR spectra of the selected samples. Right insert: the half width at half maximum (HWHM) of the L1 line versus the Cu-exchange level for the CuNH_4_-MOR-10-*XY* (squares) and CuH-MOR-10-*XY* (circles) samples. Open and closed symbols correspond to the conventional and MW-assisted treatment, respectively; semi-closed symbols represent the starting materials. Dashed lines correspond to the linear fitting of all the data for the samples with Cu-exchange level below 60%.

Regarding the above, note that XRD measurements provided us with information about the idealized topological structure of the framework; any kinds of defects are not reflected. Local distortions of the surrounding of Al atoms can be revealed by magic angle spinning nuclear magnetic resonance (MAS NMR). The selected spectra are shown in Figure 2 (see left insert) with an example of the spectrum decomposition for H-MOR-10. The ^27^Al MAS NMR spectra for all the studied samples are given in Supplementary Materials (Figures S3 and S4).

The ^27^Al MAS NMR spectrum for NH_4_-MOR-10 represents one line L1, the narrowest among the all studied samples. The annealing of NH_4_-MOR-10 at 300 °C (to produce H-MOR-10) led to the appearance of two additional lines at 0 and 38 ppm, Gaussian lines G1 and G3, respectively, which corresponded to six- and so-called “five”-coordinated Al, see Figure 2. This shows that, despite that the whole crystal structure is preserved, there was some local destruction of the zeolite framework, with a total amount of out-of-lattice Al atoms of about 10%. Nevertheless, even the first ion-exchange procedure led to the disappearance of the six-coordinated Al (line G2 in Figure 2); however, the G3 line was always observed in the CuH-MOR-10-*XY* series and was almost unchanged. The framework observed for the recovery of protonated zeolites after a conventional ion exchange with copper is much more spectacular for mordenites with a low Si/Al ratio (e.g., Si/Al = 5, see [25]). In the present study, for mordenites with a nominal value of Si/Al = 10, we observed that the MW-assisted procedure also helped eliminate the six-coordinated Al. In contrast to this, the framework defects, which revealed themselves in the ^27^Al spectra as “five”-coordinated Al, were accumulated with an increase in the number of the ion-exchange procedures, see Supplementary Materials (Figures S3 and S4). This effect was not very pronounced, but is worth being mentioned.

### 2.3. Thermal Analysis

**Figure 3:**
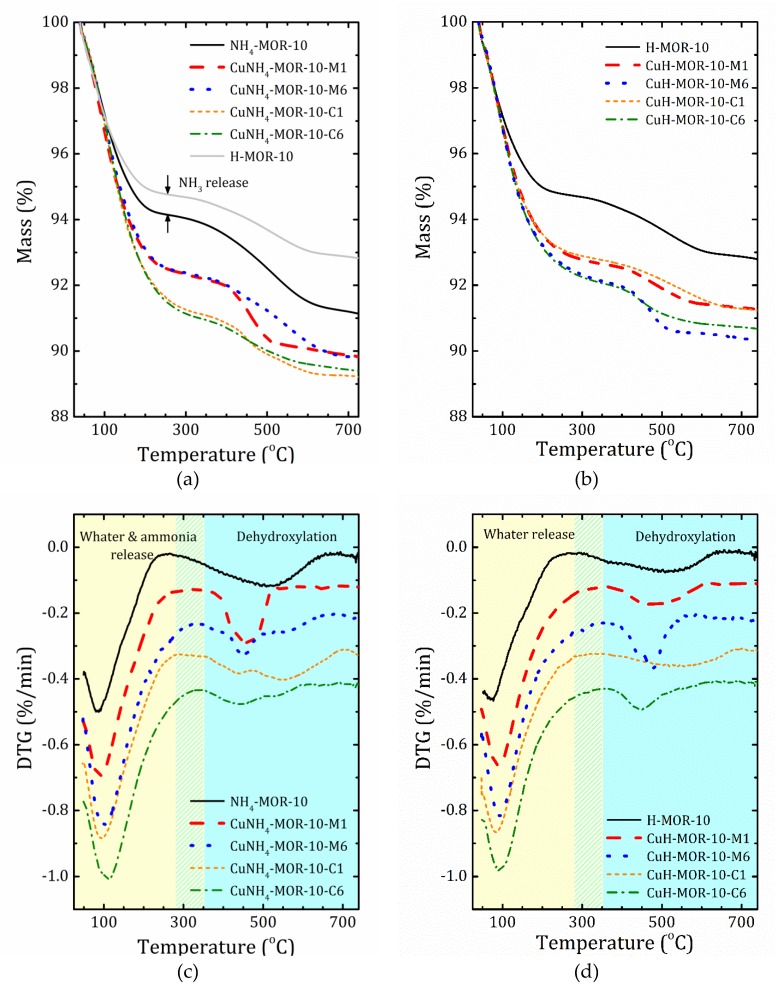
Thermogravimetric (TG) (**a**,**b**) and derivative thermogravimetric (DTG) (**c**,**d**) profiles for the starting samples and after the first and six ion-exchange procedures synthetized from the ammonia (**a**,**c**) and proton (**b**,**d**) forms. To guide to the eye, the DTG curves for different samples are shifted down with a step 0.1%/min.

Thermogravimetric (TG) profiles of selected samples together with the derivative thermogravimetric (DTG) curves are shown in Figure 3. The results for all the studied samples are given in Supplementary Materials (Figures S5 and S6). As one can see, the mass loss occurred in two main steps. The first step was due to water desorption, while the second one was caused by thermal decomposition of hydroxyl groups. Based on the DTG curves decomposition, we introduced the *T*_0_ temperature to separate these two steps. The mass loss and *T*_0_ values are listed in Table 3.

### 2.4. Cu2p X-ray Photoelectron Spectroscopy (XPS) Analysis

The valence state of copper ions was probed by X-ray Photoelectron Spectroscopy (XPS). The Cu2p spectra for all samples and their analysis are given in Supplementary Materials (Figure S7 and Table S1). The XPS studies indicated that, in all the studied samples, copper ions are in the Cu^2+^ state. It should be noted that all samples, throughout the process of multiple ion exchange using both MW and the conventional method, retained the typical blue color of Cu^2+^. The deconvolution of Cu2p_3/2_ spectral line shows that the binding energy that corresponds to the main peak with the Cu2p_3/2_ satellite line is about 933.6 eV. This can be attributed to the Cu^2+^ ion bound to ligands by covalent bonds [24,29]. An additional peak of low intensity that is shifted towards higher binding energy values (near 935 eV) can be associated with Cu^2+^ coordinated by water [27].

## 3. Discussion

One of the processes studied in the present work was the copper accumulation in mordenite due to exchange procedures done in two different ways. As seen from Table 1, after the first ion exchange treatment by both methods, copper ions did not substitute the initial compensating cations completely: the copper-exchange level did not exceed 36% for the two methods and both systems. Nevertheless, after multiple ion exchange procedures, the MW treatment proved itself to be more effective, especially for CuH-MOR-10-M*Y* series. However, in contrast to the previously studied copper-exchanged mordenites obtained from Na-MOR-6.5 [27], even six subsequent MW-assisted ion exchange procedures did not lead to an over-exchange. This discrepancy between the ammonia and proton forms of mordenite, unlike sodium, is an interesting fact, but its cause is not yet clear; one of the reasons, as we can assume, is due to the different Si/Al ratio and its effect on the exchange properties of mordenite. As expected, zeolites with the same framework topology, but with different framework charges, demonstrate quantitative differences in the exchange behavior [29]. Less obvious, the charge density of the zeolite framework also affects ion exchange selectivity [30]. These effects are especially pronounced for uni–divalent cations exchange. Since the charge of divalent cations is balanced by two negative framework sites, the selectivity and maximum level of exchange in zeolites with low aluminum content depend mainly on the separation distances of the framework charge sites [30]. However, a detailed study on similar dependencies of the role of various counter-cations on the exchange of the target cation, and the role of the composition of zeolite in such processes, is beyond the scope of this present work and will be done separately. Another interesting observation was that for all the systems studied in this work, obtained both by conventional and MW-assisted exchange methods, the copper exchange stopped at approximately 70%, as was previously detected for CuNa-MOR-6.5-C*Y*, prepared by the conventional method in [27].

**Figure 4:**
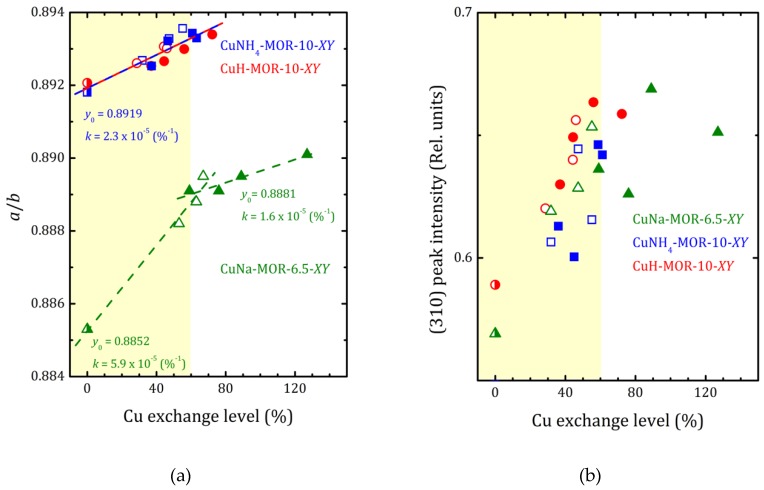

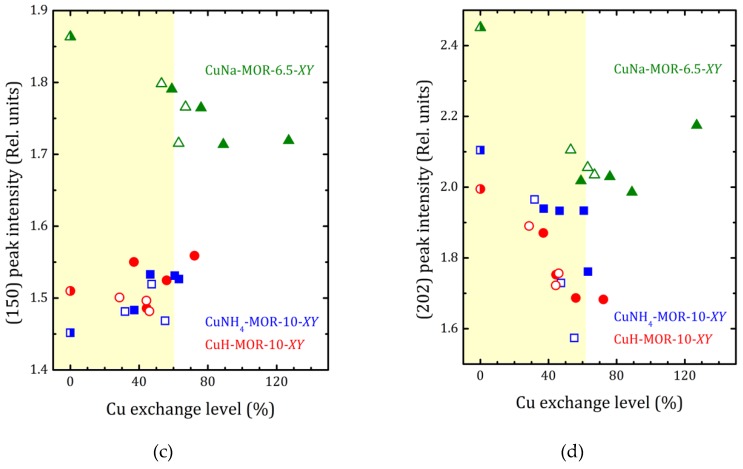
The *a*/*b* ratio (**a**) and normalized (310), (150), and (202) peak intensities ((**b**–**d**), respectively) versus copper-exchange level for the CuNH_4_-MOR-10-*XY* (squares), CuH-MOR-10-*XY* (circles) and CuNa-MOR-6.5-*XY* (triangles) series; open and closed symbols correspond to the conventional and MW-assisted treatment, respectively; semi-closed symbols correspond to the starting materials. Dashed lines show the linear fitting (*y* = *y*_0_ + *kx*) of all the *a*/*b* data for CuNH_4_-MOR-10-*XY* and CuH-MOR-10-*XY* together; for CuNa-MOR-6.5-*XY* the data for the samples obtained by conventional and MW-assisted treatments are fitted separately; corresponding fitting parameters *y*_0_ and *k* are given. The data for CuNa-MOR-6.5-*XY* are taken from [27] (adapted with permission from Ref. [27] Copyright 2017 Elsevier Inc.).

As it follows from XRD analysis, the copper loading led to increasing the *a* to *b* lattice parameters ratio that reflects the compression of the main mordenite channel. The *a*/*b* values versus the copper-exchange level for all the studied samples are plotted in Figure 4 together with the data for CuNa-MOR-6.5-*XY* series from [27]. As one can see from Figure 4, for both CuNH_4_-MOR-10-*XY* and CuH-MOR-10-*XY*, the copper exchange led to increasing the *a*/*b* ratio that can be almost perfectly fitted by linear dependence. Moreover, neither the nature of the initial cation nor the preparation method has any significant effect on the slope of the line, which is essentially lower in comparison with that one for CuNa-MOR-6.5-C*Y*. The weakening of the effect of Cu imbedding on the deformation of the mordenite lattice was related to the lower absolute copper content at the same copper-exchange level (due to higher Si/Al ratio). We note that the change in the slope after 70% of the copper-exchange level observed in CuNa-MOR-6.5-M*Y* series can be attributed to the creation of [Cu-O-Cu]^2+^ complexes formed due to MW irradiation [27].

XRD patterns presented in Figure 1 and Figures S1 and S2 show that the ammonium- or proton-to-copper exchange led to some changes in relative intensities of diffraction peaks. Figure 4b–d show the changes in intensities of selected peaks, namely (310), (150), and (202), that took place with copper loading. To analyze their intensities, they were normalized to the intensity of the peak (330), which varied much less than others from sample to sample (see Figure 4). Besides these, for both forms, ammonium and proton, it can be seen that the exchange for copper changed the intensity of the peaks (110), (020), and (200). Schematic representation of these planes is given in Supplementary Materials, Figure S8. The changes are fairly constant with increasing copper concentration and does not do beyond the general trend. This is quite expected for ion-exchange zeolites, and the effect is mainly due to differences in nature, number, and position of extra-framework cations. This indicates that Cu^2+^ ions occupy similar cationic positions in the channels of mordenite, regardless of the exchange method used. A more detailed study of the structure is undoubtedly of interest, as it would provide valuable information on the local structure rearrangement caused by ion-exchange procedure, as it was observed in Ag- and Fe-exchanged mordenite [31], and its results will be published elsewhere.

**Figure 5:**
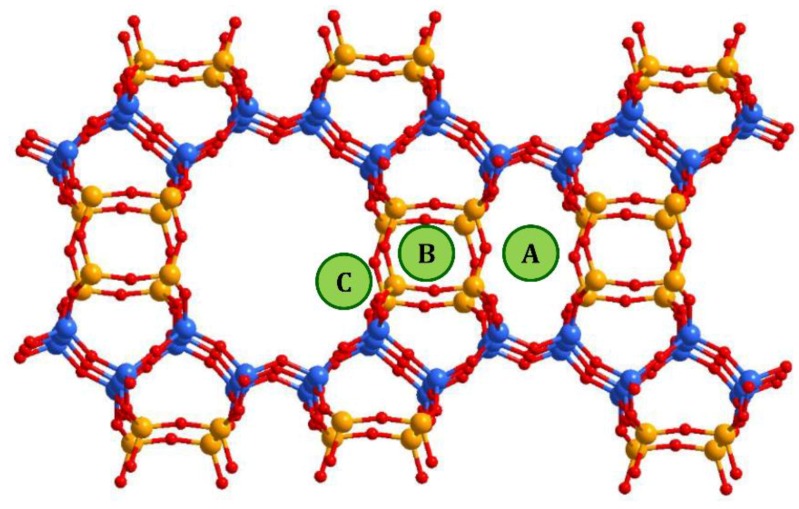
Possible cationic sites in the mordenite framework. Blue balls correspond to Si atoms in T1 and T2 sites of the mordenite lattice, orange balls are those Si atoms that can be substituted by Al (T3 and T4 sites).

In mordenite, counter-cations can occupy different positions. From general considerations, cations tend to be located at positions that are more stable or have minimal energy, which includes optimal values for cation/framework distances, the distance between cation and water molecules, between water molecules and the framework, minimal repulsion between guest species, etc. Referring to the studied systems, and taking into account the results of previous studies, the following possible cationic sites, shown in Figure 5, were proposed in [32]: A—inside the small mordenite channel; B— in the side pocket; and C—inside the main channel near the side pocket. Here we did not proceed to a deeper description since the methods used in the present study did not allow us to distinguish counter cations localized, for example, in different places within the main channel; only a rough analysis can be done. Thus, despite the fact that copper ions do not have exact crystallographic positions, the behavior of the intensities of the XRD peaks that reflects the influence of the copper atoms can be summarized as follows. The decrease in the intensity of the peak (202) (as well as that one of (020) and (200)) occurred due to the displacement of [NH_4_]^+^ (or H^+^) cations, originally located in small mordenite channels (site A in Figure 5) that occur under the action of the ion-exchange procedure. The above-mentioned cations leave their sites, but when entering Cu^2+^ cations do not replace them, preferring to settle in the main channel near the entrance to the side pocket, the C-site. Note, that the planes (202) and (310) intersect in the side pocket entrance area. That is, the localization of the cation in this site should have a strong influence.

The difference in behavior of (150) peak intensity between CuNH_4_-MOR-10-*XY* and CuH-MOR-10-*XY* series and the data from [27] for the CuNa-MOR-6.5-*XY* series means that the exact position of the copper ions in the main channel of mordenite with Si/Al = 10 and Si/Al = 6.5 were different. As Al can occupy T3 and T4 sites marked by orange in Figure 5, with Si/Al increasing, the occupancy of these two sites by Al decreases and the total number of cationic sites decreases.

A slight discrepancy in the peak intensities observed for CuNH_4_-MOR-10-*XY* and CuH-MOR-10-*XY* samples prepared by different methods may be due to the fact that copper ions can occupy different sites in the mordenite lattice, which corresponds to the further discussion of the results of the thermogravimetric analysis (TGA). The latter was also consistent with the ^27^Al MAS NMR study. The introduction of copper into the studied zeolite did not shift the main L1 line, but led to its broadening, which is a typical effect of paramagnetic ions and implicitly confirms that copper ions are in the Cu^2+^ state. The right insert in Figure 2 shows the half width at half maximum (HWHM) of the L1 line plotted versus the copper-exchange level. This parameter does not carry direct information about the preparation method. It can be fairly well fitted with a straight line; a noticeable deviation occurred only when the copper-exchange level exceeded 60%, which may indicate a redistribution of copper ions as compared with other ion exchange positions, and confirms, once again, the presence of a threshold exchange level before and after which the behavior of cations in the zeolite changes.

For CuNa-MOR-6.5-M*Y*, where the MW treatment possibly led to the creation of [Cu-O-Cu]^2+^ species [28], changes in the intensities of diffraction peaks at high copper loading (>60%) reflect that these complex cations occupy new sites and/or, perhaps, their appearance led to a redistribution of Cu^2+^.

Valuable information on differences in the accessibility of ion-exchanged sites in the CuNH_4_-MOR-10-*XY* and CuH-MOR-10-*XY* series can be provided by TGA analysis. Compared with the data of CuNa-MOR-6.5-*XY* [27], in the ammonia and protonated series, firstly, the high temperature peak of mass loss always presents and is rather important; secondly, the temperature of the first step is essentially shifted towards a lower temperature.

According to the XRD analysis, in NH_4_-MOR-10, ammonium ions were in a small mordenite channel and left it during substitution with copper. This means that before the copper is loaded, the main channel is almost empty: water molecules are weakly held by the zeolite framework due to a high Si/Al ratio (and, therefore, low polarization). Copper, entering into the main channel, is coordinated by water that results in an increase in mass loss due to water release at the first step; see Figure 3 and Table 3. In the CuH-MOR-10-*XY* series, an additional decrease in the water content could be related with partial destruction of the zeolite framework.

Turning to a comparison of ammoniated and protonated forms of mordenite used for copper exchange, it should first be emphasized that the initial step of water desorption was also accompanied by ammonia release due to thermal decomposition of [NH_4_]^+^, which ends at about 250 °C. Unfortunately, the close mass values of NH_3_ and H_2_O make them difficult to distinguish. Nevertheless, the quantity of ammonia emission can be roughly estimated from the difference between the TGA curves for NH_4_-MOR-10 and H-MOR-10 at the first step of mass loss, see Figure 3a. The second step of mass loss at temperatures above *T*_0_, which was between 258 and 364 °C, depending on the sample, see Table 2, was caused by dehydroxylation processes. From this perspective, the second step of the TGA curves for NH_4_-MOR-10 and H-MOR-10 should be the same. Indeed, the profiles looks quite similar; the difference in mass loss, that is the difference in the number of hydroxyl groups, could be caused by the fact that the preparation of H-MOR-10 is a static calcination process (two hours at 300 °C), whereas during the TGA study, the NH_4_-MOR-10 sample experienced a dynamic thermal exposure. In other words, the main difference between the ammonia and protonated series was the amount (and possibly the number of types) of hydroxyl groups that should influence ion exchange since hydroxyls in the protonated form may occupy several ion-exchangeable sites making others accessible, which were initially less energetically favorable. The presence of the so-called “five”-coordinated Al may also lead to a redistribution between more and less favorable ion-exchanged sites.

In the ammonia samples, after the completion of the ion-exchange procedure, part of [NH_4_]^+^ was replaced by Cu^2+^, but the remaining ammonium cations thermally decomposed at temperatures below 300 °C, forming new proton centers. These former CuNH_4_-MOR-10-*XY* samples at temperatures above 300 °C are converted into “CuH-MOR-10-*XY*”, but with different numbers (and types) of hydroxyl groups, which are now determined by the previous location of the residual ammonium cations in the mordenite structure after ion exchange.

Another important point, as can be seen from Figure 3, is that the presence of copper affects the formation of a certain form of hydroxyls; as the concentration of copper increased, hydroxyl groups that decompose at high temperatures disappeared first, and the relative number of low temperature ones increased. This implicitly points out that the high temperature hydroxyl groups in H-MOR-10 after the ion-exchange procedure may have been replaced by copper cations.

## 4. Materials and Methods

Copper-exchanged zeolites were prepared from NH_4_^+^- and H^+^-mordenites with nominal Si/Al atomic ratios (AR) equal to 10. The NH_4_^+^-mordenite was supplied by Zeolist Int. (Product CBV21A). The proton form was obtained by the calcination of NH_4_^+^ mordenite at 300 °C for 2 h. The calcination temperature was chosen on the basis of thermogravimetric analysis of the NH_4_^+^ mordenite: the temperature at which the deammoniation process had finished but the dehydroxylation had not started yet, see Section 2.3. The ammonia and proton forms were labeled as NH_4_-MOR-10 and H-MOR-10, respectively.

In order to prepare copper-exchange samples, the starting material (NH_4_-MOR-10 or H-MOR-10) was treated in 0.05 M CuSO_4_ aqueous solution, taking the volume with the twofold excess of copper amount in the solution to the estimated ion-exchange capacity of mordenite. The conventional copper ion exchange was carried out under stirring at room temperature for 1 day. For the MW-assisted ion-exchange procedure, the mixture of zeolite and CuSO_4_ aqueous solution (with the same concentration of solution and the same zeolite-solution ratio) was heated at 100 °C in a Synthos 3000 Anton Paar microwave oven at 1400 W for 2 h. The exchange process was repeated several times (up to six) to increase the copper content. The samples obtained by both methods were filtered, thoroughly washed, and dried at room temperature overnight. The samples obtained from the ammonia and proton forms were labeled as CuNH_4_-MOR-10-*XY* and CuH-MOR-10-*XY*, respectively, with *X* = C or M for the conventional (C) or MW (M) ion-exchange preparation method, correspondingly; *Y* = number of copper-exchange procedures; digit 10 corresponds to the Si/Al ratio. The main information concerning the exposure, which the studied samples underwent during preparation treatment, is summarized in Table 4.

XRD analysis was done using a Bruker D8 DISCOVER (Bruker Corporation, Billerica, MA, USA) diffractometer with CuK_α_ long-focused X-ray sealed tube (scan range: from 5.0017 to 60.0041 2-theta degree, step width: 0.0202 degree). Quantification was completed using the DIFFRAC.SUITE software package.

The chemical composition of the samples before and after the ion-exchange procedures was explored by ICP-OES using Shimadzu ICPE-9000 (Shimadzu Corporation, Kyoto, Japan). The valence state of copper ions was probed by XPS analysis carried out using a Combined Auger, X-ray and Ultraviolet Photoelectron spectrometer Thermo Fisher Scientific ESCAlab 250Xi with monochromatic AlK_α_ radiation (photon energy 1486.6 eV (Thermo Fisher Scientific, Waltham, MA, USA) at room temperature in ultra-high vacuum with pressure of the order of 1 × 10^-9^ mbar.

The water content and products of the samples decomposition were determined through the use of thermal analysis using a Netzsch STA 449 F1 Jupiter coupled with a quadrupole mass spectrometer QMS 403 Aëolos (Netzsch-Gruppe, Selb, Germany). The analysis was done in the temperature range from 40 to 700 °C at a heating rate of 10 °C/min in argon stream at the rate of 90 mL/min.

To probe the regularity of the zeolite framework the magic angle spinning nuclear magnetic resonance (MAS NMR) method was used. ^27^Al MAS NMR spectra were recorded by applying the zg30 pulse sequence at 104.29 MHz with rotation frequency 8 kHz. Chemical shifts were determined relative to the AlCl_3_ solution as an external standard. NMR measurements were carried out at room temperature using Bruker WB Avance III (400 MHz) spectrometer (Bruker Corporation, Billerica, MA, USA).

## 5. Conclusions

The main conclusions of the work can be formulated as follows:

For both of the studied initial forms of mordenite, neither MW-assisted nor conventional methods destroy the zeolite framework. As compared with the sodium form of mordenite, the exchange of copper in ammonia and protonated forms of mordenite behaves differently but all copper is in Cu^2+^ form. The applied ion-exchange processes do not lead to an over-exchange, as the level of copper exchange is limited to 70%.

The exchange of the initial counterions for Cu^2+^ ions leads to a linear increase in the *a*/*b* ratio with an increase in the copper exchange level. This means that all copper cations are ion-exchangeable and enter the main mordenite channel. For CuH-MOR-10-M6, with the greatest amount of copper reaching 70% of the degree of exchange, changes in the width of the ^27^Al MAS NMR line are observed. This effect can be interpreted as the possible appearance of a new form of copper, or the redistribution of these copper ions along ion-exchangeable sites when the threshold value is reached.

When heated, there is a loss of water, which is accompanied by the simultaneous release of NH_3_ for the CuNH_4_-MOR-10-*XY* samples. This process ends in the range of 260–360 °C. With further increases in temperature, the mass loss is due to dehydroxylation that occurs in a complex way. In copper-exchanged samples, protons (hydroxyl groups) with a higher temperature of thermal decomposition are selectively replaced. There is a correlation between the energy of the exchange for copper and the energy of decomposition of various ammonium groups and, accordingly, the dehydroxylation of proton groups formed from non-exchanged ammonium cations.

Summing up the above, one can conclude that MW-assisted treatment has proven itself as an efficacious method for the synthesis of copper-exchanged mordenites, which not only significantly reduces preparation time but leads to a systematically higher Cu-exchange level.

## Figures and Tables

**Table 1:** Chemical composition of the bulk samples as determined from inductively coupled plasma-optical emission spectroscopy (ICP-OES) data.

Sample	ICP-OES		
	Cu/Al	Si/Al	Cu-Exchange Level (%)
NH_4_-MOR-10	–	8.5(1)	–
CuNH_4_-MOR-10-M1	0.19(1)	8.4(1)	37.2(1)
CuNH_4_-MOR-10-M2	0.23(1)	8.3(1)	46.5(1)
CuNH_4_-MOR-10-M3	0.32(1)	8.2(1)	63.2(1)
CuNH_4_-MOR-10-M6	0.30(1)	8.0(1)	60.7(1)
CuNH_4_-MOR-10-C1	0.16(1)	8.3(1)	31.8(1)
CuNH_4_-MOR-10-C3	0.24(1)	8.1(1)	47.3(1)
CuNH_4_-MOR-10-C6	0.27(1)	8.2(1)	55.1(1)
H-MOR-10	–	8.6(1)	–
CuH-MOR-10-M1	0.18(1)	8.6(1)	37.0(1)
CuH-MOR-10-M2	0.22(1)	8.5(1)	44.4(1)
CuH-MOR-10-M3	0.28(1)	8.5(1)	56.0(1)
CuH-MOR-10-M6	0.36(2)	8.4(1)	72.2(1)
CuH-MOR-10-C1	0.14(1)	8.5(1)	28.6(1)
CuH-MOR-10-C3	0.22(1)	8.3(1)	44.3(1)
CuH-MOR-10-C6	0.23(1)	8.5(1)	46.0(1)

**Table 2:** Lattice parameters, unit cell volume, and *a*/*b* ratio for the studied samples before and after the ion-exchange procedure.

Sample	Lattice Parameters (Å)			Unit Cell Volume (Å^3^)	*K* = *a*/*b*
	*a*	*b*	*c*		
NH_4_-MOR-10	18.167(2)	20.371(1)	7.4941(4)	2773(2)	0.8918(1)
CuNH_4_-MOR-10-M1	18.204(2)	20.396(1)	7.5020(4)	2785(2)	0.8925(1)
CuNH_4_-MOR-10-M2	18.186(2)	20.360(1)	7.4956(4)	2775(2)	0.8932(1)
CuNH_4_-MOR-10-M3	18.185(2)	20.357(1)	7.4944(4)	2774(2)	0.8933(1)
CuNH_4_-MOR-10-M6	18.209(2)	20.381(1)	7.5001(4)	2783(2)	0.8934(1)
CuNH_4_-MOR-10-C1	18.183(2)	20.369(1)	7.4959(4)	2776(2)	0.8927(1)
CuNH_4_-MOR-10-C3	18.189(2)	20.362(1)	7.4976(4)	2777(2)	0.8933(1)
CuNH_4_-MOR-10-C6	18.192(2)	20.359(1)	7.4979(4)	2777(2)	0.8936(1)
H-MOR-10	18.166(2)	20.364(1)	7.4917(4)	2771(2)	0.8921(1)
CuH-MOR-10-M1	18.196(2)	20.387(1)	7.4985(4)	2782(2)	0.8925(1)
CuH-MOR-10-M2	18.171(2)	20.356(1)	7.4929(4)	2772(2)	0.8927(1)
CuH-MOR-10-M3	18.184(2)	20.363(1)	7.4957(4)	2776(2)	0.8930(1)
CuH-MOR-10-M6	18.194(2)	20.365(1)	7.4948(4)	2777(2)	0.8934(1)
CuH-MOR-10-C1	18.169(2)	20.355(1)	7.4918(4)	2771(2)	0.8926(1)
CuH-MOR-10-C3	18.181(2)	20.358(1)	7.4937(4)	2774(2)	0.8931(1)
CuH-MOR-10-C6	18.180(2)	20.358(1)	7.4938(4)	2774(2)	0.8930(1)

**Table 3:** Mass loss (%) due to water (and ammonia) release (below *T*_0_) and due to dehydroxylation (above *T*_0_).

Sample	Mass Loss (%) in the Temperature Range:		*T*_0_ (°C)
	40 °C *T* *T*_0_	*T*_0_*T* 740 °C	
NH_4_-MOR-10	5.86	3.02	258
CuNH_4_-MOR-10-M1	7.64	2.57	350
CuNH_4_-MOR-10-M2	8.63	2.02	356
CuNH_4_-MOR-10-M3	11.10	1.15	364
CuNH_4_-MOR-10-M6	8.77	2.04	307
CuNH_4_-MOR-10-C1	7.81	2.42	283
CuNH_4_-MOR-10-C3	8.44	1.93	323
CuNH_4_-MOR-10-C6	8.97	1.65	325
H-MOR-10	5.26	1.95	270
CuH-MOR-10-M1	7.28	1.46	325
CuH-MOR-10-M2	7.15	1.44	336
CuH-MOR-10-M3	7.64	1.59	331
CuH-MOR-10-M6	7.76	1.93	322
CuH-MOR-10-C1	7.19	1.58	331
CuH-MOR-10-C3	7.04	1.51	340
CuH-MOR-10-C6	7.82	1.53	355

**Table 4:** Classification of the studied copper-exchanged samples according to the undergone exposure. Processing time is indicated for one stage of the exchange. Every time the exchange was repeated, the exposure time increased.

Sample Name	Initial Cation Form	Pretreatment	Ion Exchange Procedure
NH_4_-MOR-10	[NH_4_]^+^	No	No
H-MOR-10	H^+^	120 min at 300 °C	No
CuNH_4_-MOR-10-M*Y*	[NH_4_]^+^	No	MW at 100 °C
CuNH_4_-MOR-10-C*Y*	[NH_4_]^+^	No	conventional at 20 °C
CuH-MOR-10-M*Y*	H^+^	120 min at 300 °C	MW at 100 °C
CuH-MOR-10-C*Y*	H^+^	120 min at 300 °C	conventional at 20 °C

## Supplementary Materials

The following are available online. Supplementary Materials include the XRD patterns, TG/DTG curves, ^27^Al MAS NMR spectra and XPS Cu2p_3/2_ peaks with their deconvolution for the studied sample as well as an image of the *c*-doubled mordenite unit cell with selected (hkl) planes.
