# Supplementary material for: Comprehensive Analysis of the Copper Exchange Implemented in Ammonia and Protonated Forms of Mordenite Using Microwave and Conventional Methods
Source: Molecules. 2019 Nov 20;24(23):4216. doi: 10.3390/molecules24234216 (PMC6930670; doi:10.3390/molecules24234216)
Supplement: Supplementary file 1 [file molecules-24-04216-s001.pdf]

Supplementary materials

Comprehensive analysis of the copper exchange implemented in ammonia and protonated forms of mordenite using microwave and conventional methods

Marina G. Shelyapina[1,*], Ekaterina A. Krylova[1], Yurii M. Zhukov[1], Irina A. Zvereva[1], Inocente Rodriguez-Iznaga[2], Vitalii Petranovskii[3], Sergio Fuentes-Moyado[3]

[1]Saint-Petersburg State University, 7/9 Universitetskaya nab., St. Petersburg 199034, Russia
[2]Instituto de Ciencias y Tecnología de Materiales (IMRE) – Universidad de La Habana, Zapata y G, s/n La Habana 10400, Cuba
[3]Centro de Nanociencias y Nanotecnología, Universidad Nacional Autonoma de Mexico, Ensenada 22860, Baja California, México

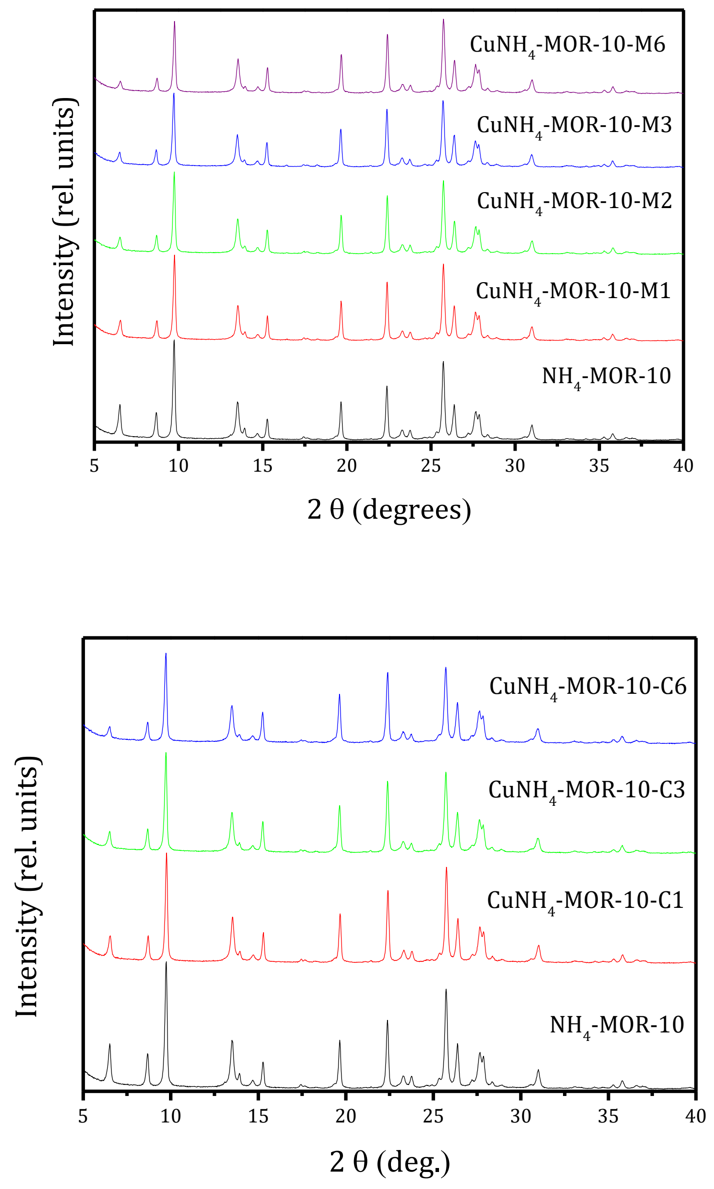

(a)

(b)

Figure S1. XRD patterns for NH_4-MOR-10 mordenite before and after copper-exchange procedure done by the MW (a) and conventional (b) methods.

* *Corresponding author*: Department of Nuclear Physics Research Methods, Saint Petersburg State University, 198504, Ulyanovskaya 3, Peterhof, Russia; e-mail: marina.shelyapina@spbu.ru

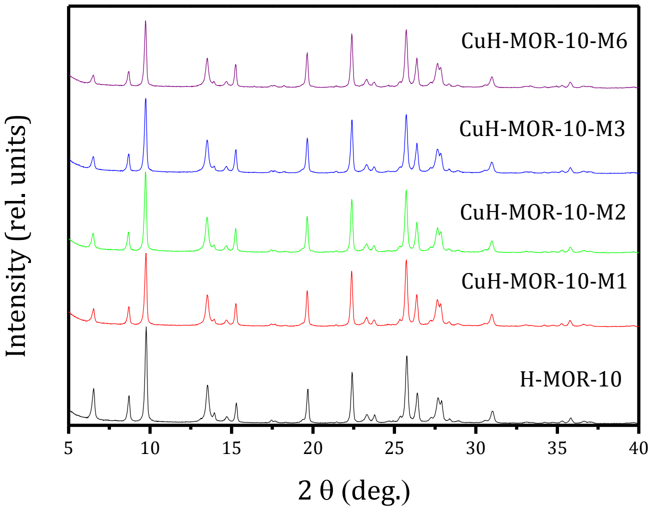

(a)

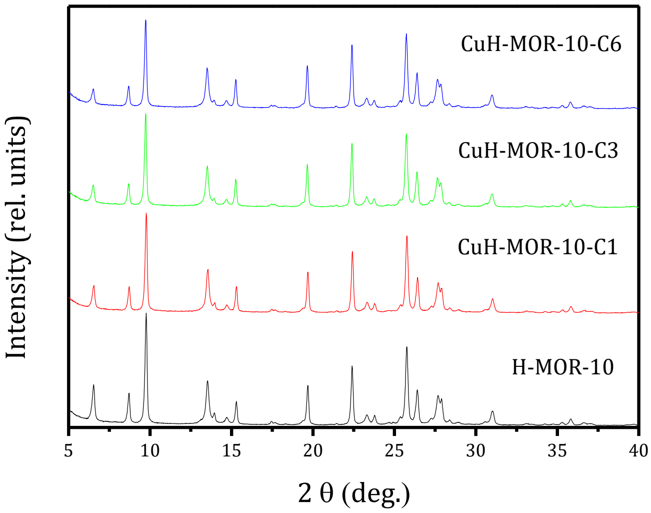

(b)

Figure S2. XRD patterns for the H-MOR-10 mordenite before and after copper-exchange procedure done by the MW (a) and conventional (b) methods.

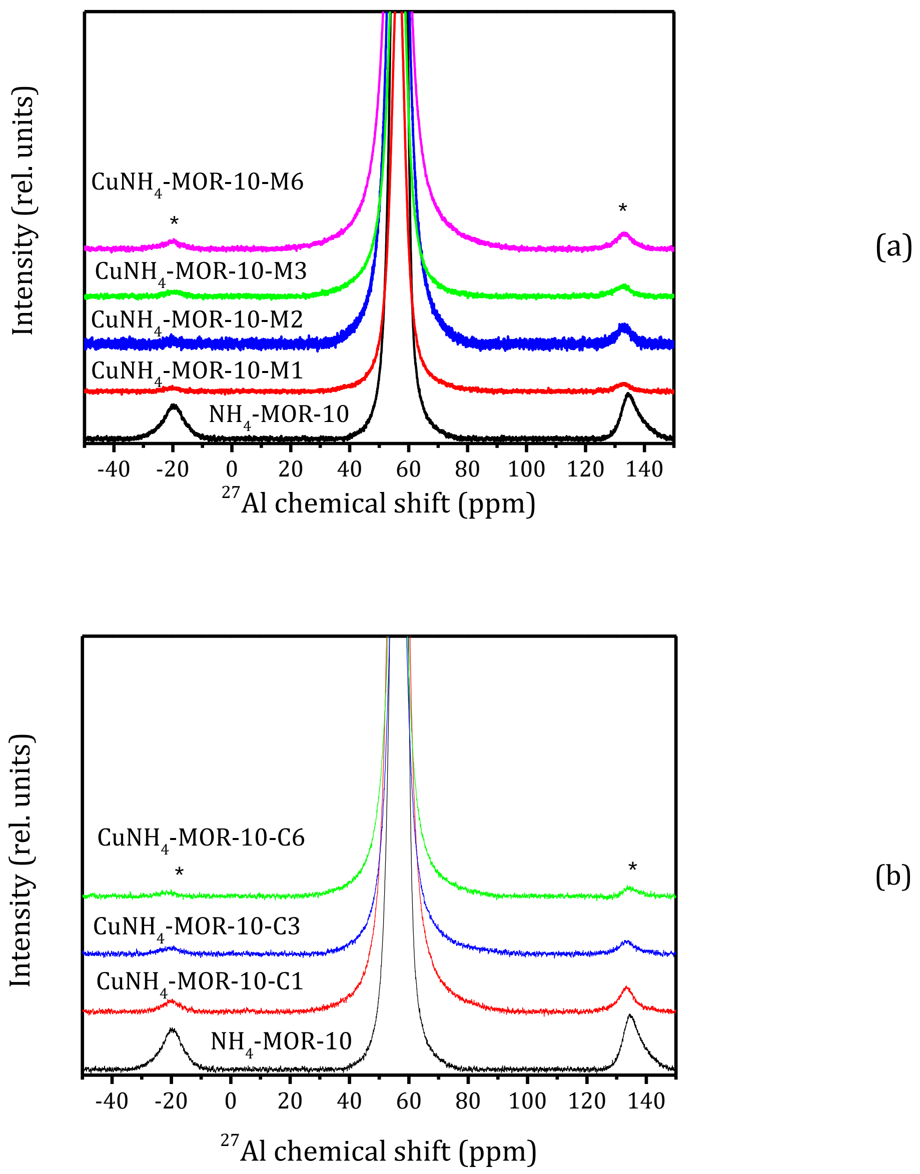

Figure S3. ^{27}Al MAS NMR spectra of the NH_4-MOR-10 mordenite before and after copper-exchange procedures done by the MW (a) and conventional (b) methods. Asterisks mark spinning sidebands.

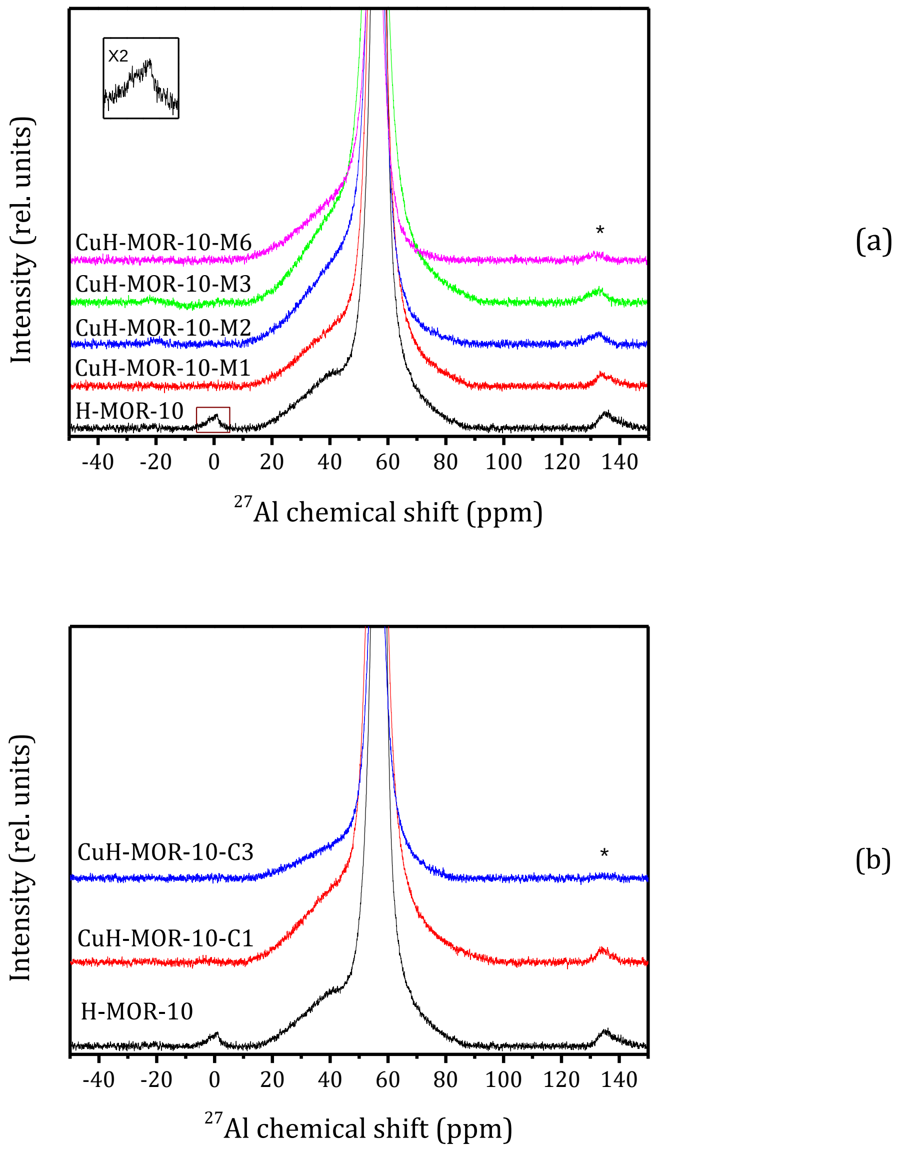

Figure S4. ^{27}Al MAS NMR spectra of the H-MOR-10 mordenite before and after copper-exchange procedures done by the MW (a) and conventional (b) methods. Asterisks mark spinning sidebands.

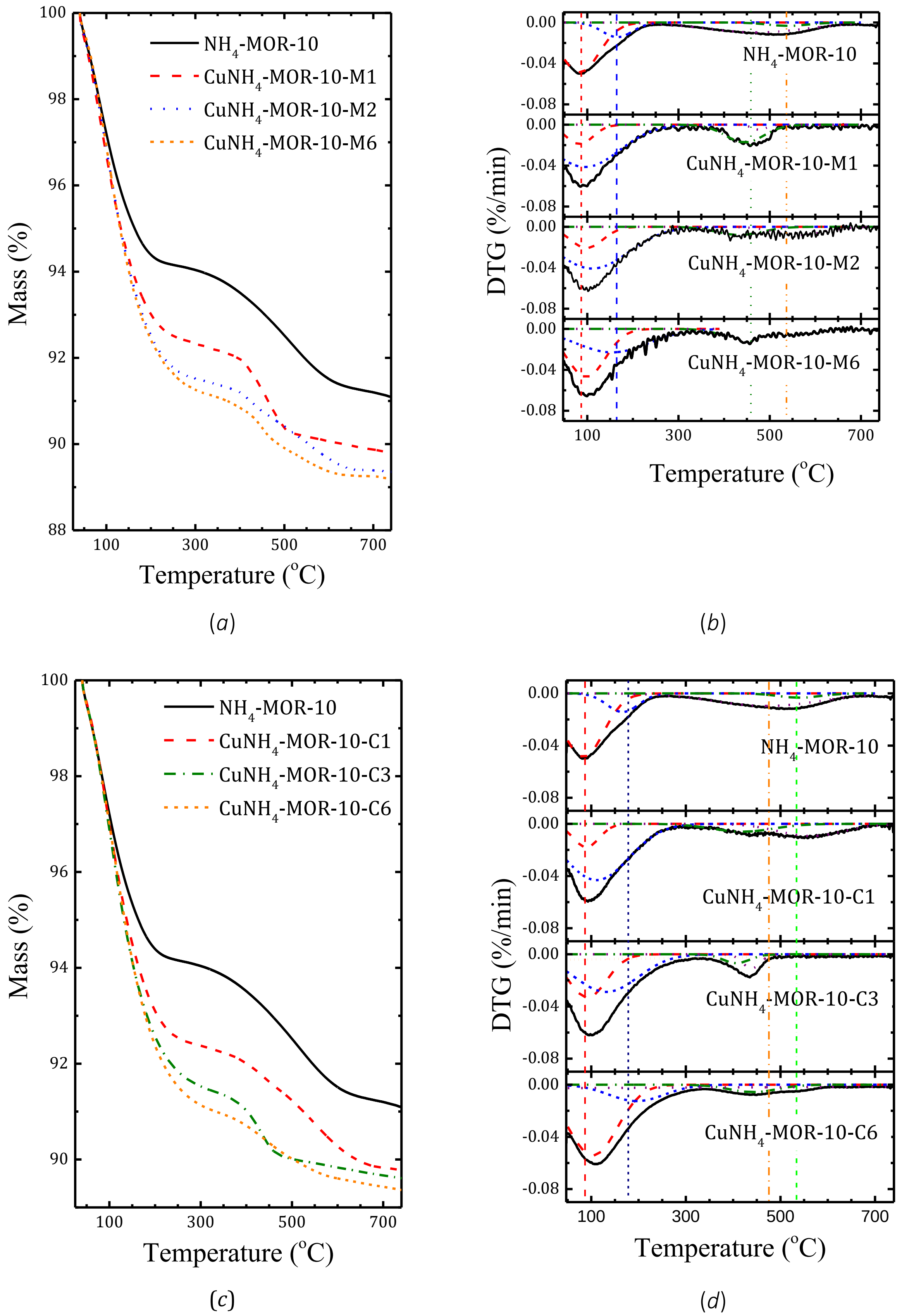

Figure S5. TG (*a*, *c*) and DTG (*b*, *d*) profiles for the studied samples synthetizes from the NH_4-MOR-10 mordenite by the MW (*a*, *b*) and conventional (*c*, *d*) methods. Vertical lines correspond to different steps of water release and are given as guides to the eye.

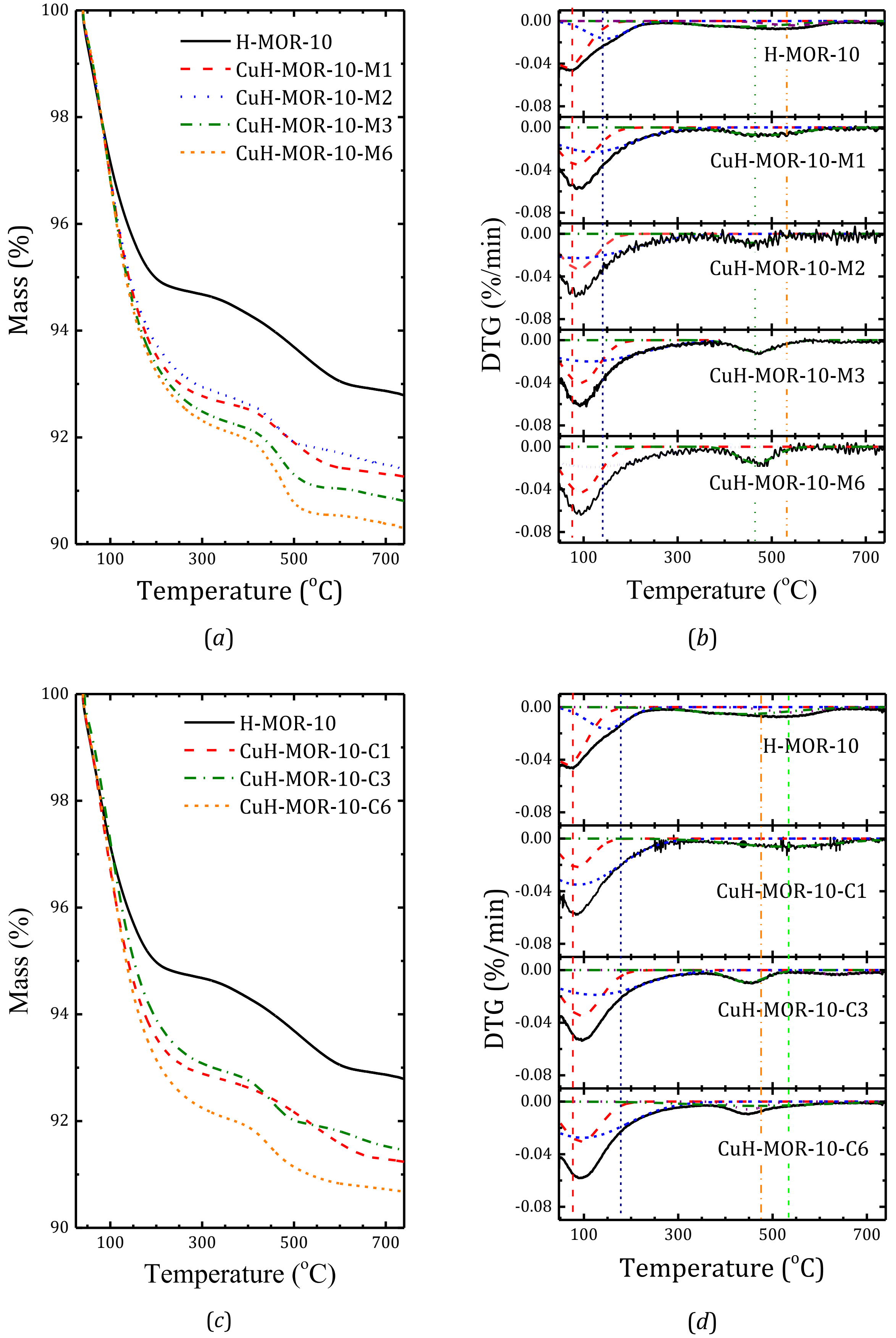

Figure S6. TG (*a*, *c*) and DTG (*b*, *d*) profiles for the studied samples synthetizes from the H-MOR-10 mordenite by the MW (*a*, *b*) and conventional (*c*, *d*) methods. Vertical lines correspond to different steps of water release and are given as guides to the eye.

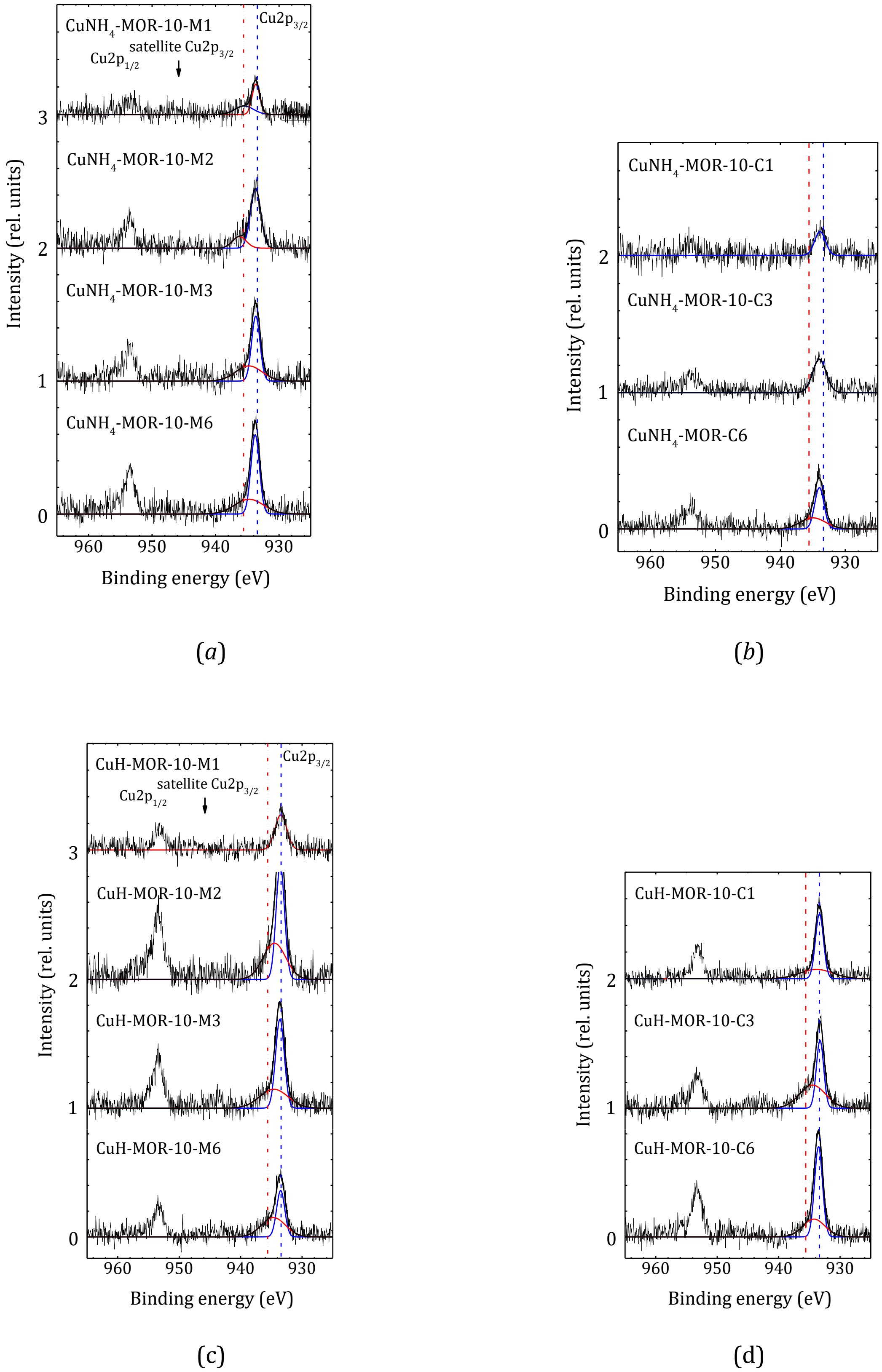

Figure S7. XPS $Cu2p_{3/2}$ and $Cu2p_{1/2}$ peaks of the studied copper-exchanged samples prepared by the MW (*a,c*) and conventional (*b,d*) methods. Vertical dashed (blue) and dot-dashed (red) lines correspond to 933.4 and 935.6 eV, respectively.

Table S1. Deconvolution of the XPS $Cu2p_{3/2}$peaks of the studied copper-exchanged samples.

Sample	Cu^{2+}(1)		Cu^{2+}(2)		Intensity ratio Cu^{2+}(1)/ Cu^{2+}(2)
	binding energy (eV)	HWHM (eV)	binding energy (eV)	HWHM (eV)	
$CuNH_4$-MOR-10-M1	933.6(4)	0.70(9)	935.5(7)	1.7(7)	2(1)
$CuNH_4$-MOR-10-M2	933.7(5)	0.96(5)	936.2(3)	1.3(4)	3.81
$CuNH_4$-MOR-10-M3	933.6(2)	0.77(3)	934.7(2)	2.4(2)	1.35
$CuNH_4$-MOR-M6	933.7(2)	0.79(2)	934.7(2)	2.7(2)	1.58
$CuNH_4$-MOR-10-C1	933.4(6)	0.98(8)	–	–	–
$CuNH_4$-MOR-10-C3	933.9(4)	1.23(4)	–	–	–
$CuNH_4$-MOR-10-C6	933.9(3)	0.85(5)	934.9(3)	2.3(3)	1.39
CuH-MOR-10-M1	933.4(6)	1.14(5)	–	–	–
CuH-MOR-10-M2	933.5(2)	0.80(2)	934.5(1)	2.2(1)	1.17
CuH-MOR-10-M3	933.6(2)	0.83(3)	934.6(2)	2.7(2)	1.44
CuH-MOR-10-M6	933.5(3)	0.78(4)	934.6(2)	2.3(1)	0.81
CuH-MOR-10-C1	933.7(1)	1.55(2)	–	–	–
CuH-MOR-10-C3	933.6(5)	0.94(5)	936.1(6)	1.2(6)	6.66
CuH-MOR-10-C6	933.9(2)	1.05(3)	–	–	–

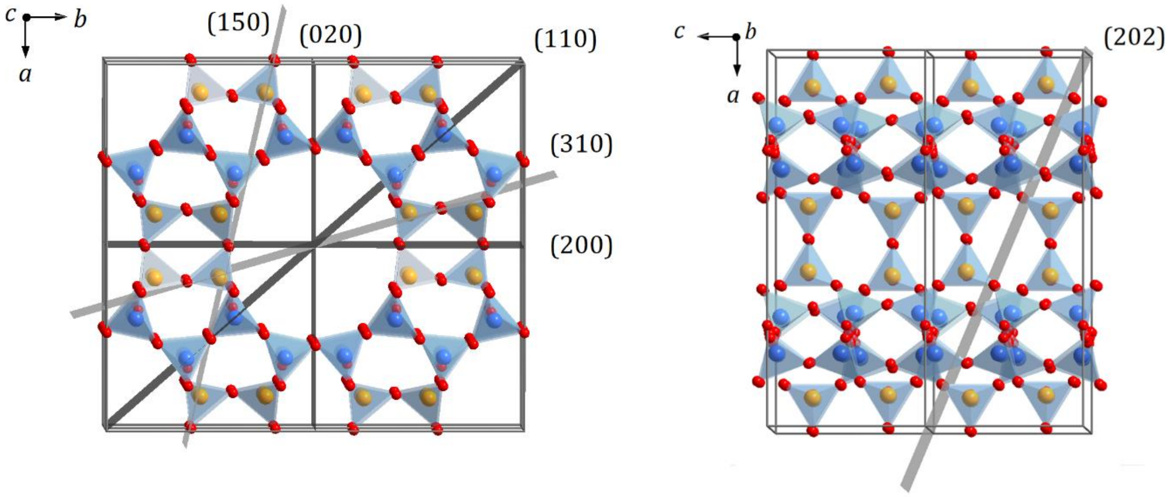

Figure S8. The c-doubled mordenite unit cell with selected (hkl) planes. Blue and yellow balls correspond to the Si and Si/Al sites, respectively.
